# Bacteriophages in Dentistry—State of the Art and Perspectives

**DOI:** 10.3390/dj7010006

**Published:** 2019-01-09

**Authors:** Liviu Steier, Silvia Dias de Oliveira, José Antonio Poli de Figueiredo

**Affiliations:** 1 Visiting Professor, Post-Graduate Program in Dentistry, Federal University of Rio Grande do Sul—UFRGS, Porto Alegre 90035-003, Brazil; lsteier@gmail.com; 2 Department of Biodiversity and Ecology, Pontifical Catholic University of Rio Grande do Sul—PUCRS, Porto Alegre 90619-900, Brazil; silviadias@pucrs.br; 3 Department of Morphological Sciences, Federal University of Rio Grande do Sul—UFRGS, Porto Alegre 90040-060, Brazil

**Keywords:** bacteriophages, dentistry, biofilm

## Abstract

Bacteriophages, viruses capable of killing bacteria, were discovered in 1915, but the interest in their study has been limited since the advent of antibiotics. Their use in dentistry is still very limited. The authors reviewed studies about bacteriophage structure, mode of action, uses in oral health, and possible future uses in dentistry associated with their possible action over biofilm, as well as the advantages and limitations of phage therapy.

## 1. Introduction

The term bacteriophage refers to viruses that are capable of destroying bacteria, or “bacteria eaters”. They are the most common biological entities on earth, at an estimated number of 10^31^ bacteriophages in the biosphere. Twort [1] and d’Hérelle [2] were the first to describe them, but it was d’Herelle who applied the term to a bacteriolytic substance that he isolated from feces. This finding leads to several studies and the creation of the “phage group”, of which Max Delbrück, James Watson, and Francis Crick were the most notable scientists [3]. 

Delbrück, in 1939, discovered a one-step process to grow bacteriophages, which, after a one-hour latent period, would multiply to produce several hundred thousands of progeny. Together with Luria, in 1943, they found a bacterium that underwent spontaneous mutations after infection by a bacteriophage until it became immune to the phage. In 1969, Delbrück, Hershey and Luria were awarded the Nobel Prize in Physiology or Medicine for their work on bacteriophages.

The discovery of antibiotics, as well as the indiscriminate use of bacteriophages to treat all types of infections, even when they were not specific to the disease, is probably the cause for the abandonment of phage therapy [4]. However, phage therapy continued to be widely practiced in the Soviet Union due to the collaboration between Felix d’Herelle and his Georgian colleagues, especially George Eliava. As a result of their studies of bacteriophages, the Institute of Vaccine and Sera in Tbilisi produced the first commercial anticholera phage preparation, which reduced the mortality due to cholera in India to 10%. D’Herelle and Eliava spent altogether 18 months in 1933 and 1934 collaborating with other scientists in Georgia [5]. D’Herelle intended to move to Tbilisi permanently, but in 1937, Eliava and his wife were killed by the Soviet regime. 

The Oswaldo Cruz Institute in Rio de Janeiro, Brazil, started the production of anti-dysentery bacteriophages in 1924 to combat dysentery in Latin American countries. Within a year, the institute had produced 10,000 vials of phages, which were sent to hospitals all over Brazil [6].

Antibiotics have been used for more than 70 years and have reduced illnesses and deaths caused by infectious diseases. The first was penicillin, and various other types followed. The emergence of bacterial resistance to the most commonly used antibiotics, a matter of major concern, may lead to the deaths of many people around the world. Such concern has renewed the interest in bacteriophages.

Bacteriophage availability for broad human application is limited, with a few exceptions [6]. A recent finding has provided evidence that bacteriophages are more virulent to bacteria in human cells than to those in bacterial cultures [7]. This may lead to further clinical research that focuses on product development and clinical application and ensures efficacy, safety, and compliance with global regulations.

As antibiotics are the standard first-line therapy against bacterial infections, the use of bacteriophages tends to have three main indications: (1) to fight infections by antibiotic-resistant bacteria; (2) to fight infections that are antibiotic resistant despite sensitivity in bacterial culture—due to poor circulation in cases of osteomyelitis and diabetic ulcers, or in the case of biofilm formation; (3) to target bacteria when antibiotics are not indicated due to, for example, patient allergies, irritable bowel problems or risk of *Clostridium difficile* infection, as well as due to concerns with excessive human and environmental exposure to antibiotics in food and agricultural applications. Some examples of cases for bacteriophage use are skin ulcers, purulent infections, methicillin-resistant *Staphylococcus aureus* (MRSA), wound prophylaxis, burns, poorly accessible infections, respiratory tract infections, urogenital tract infections and sepsis [8]. 

The use of bacteriophages in dentistry is relatively new [9,10]. The isolation of bacteriophages in oral saliva, oral tissues, dental caries and periodontal and endodontic infections may lead to a broader use. This review evaluates the mode of action of bacteriophages, describes currently available bacteriophages and points out future uses in dentistry considering the strengths and limitations of phage therapy.

## 2. Bacteriophages: Mode of Action and Types

Bacteriophages—also known as phages—are viruses that infect bacteria, where they reproduce. Their capsid, or protein shell, where the genetic content of the virus is located, may vary in shape and be icosahedral, filamentous, or head-tail. The head-tail structure seems to be unique of bacteriophages. Their genome may be of DNA, and the number of genes varies from four to several hundreds [11]. 

**Figure 1:**
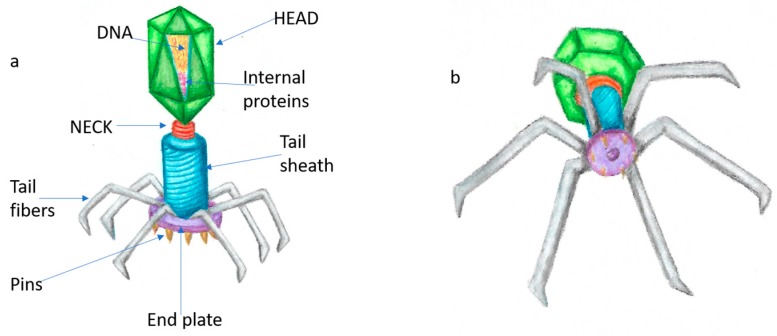
General structure of a head-tail bacteriophage—(**a**) all components; (**b**) view of the bacteriophage as if attaching to a bacterial surface.

The structure of a head-tail bacteriophage, which is the most useful for dentistry purposes, is (Figure 1):

- Head: where the capsid contains the double-strand DNA, together with internal proteins.
- Neck: connects head and tail.
- Tail: tubular structure that allows passage of DNA when in contact with bacterial surface.
- Tail fibers: proteins that attach to bacterial surface.
- End plate: contains pins that penetrate the membrane to allow phage DNA release into the host [11].

The life cycle of a bacteriophage varies according to its effect on the bacterial structure. If it leads to death of the host cell, it is called a lytic life cycle. If its DNA is copied into the host DNA, by recombining with bacterial chromosomes each time the cell divides, becoming integrated into the chromosome as a prophage, it is called a lysogenic life cycle. The bacteriophages of interest in dentistry are all lytic. They behave as typical viruses, using the cell resources to replicate and causing the cell to lyse, burst and dye in the process. 

**Figure 2:**
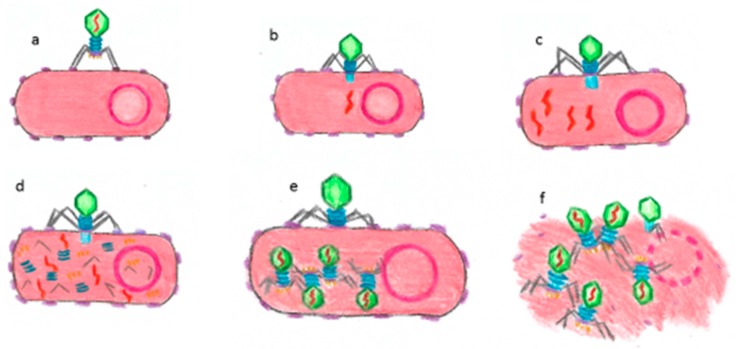
Lytic cycle of a bacteriophage: (**a**) attachment to a host receptor; (**b**) phage DNA injection; (**c**) replication of bacteriophage DNA; (**d**) biosynthesis of bacteriophage proteins; (**e**) maturation—new bacteriophage particles are assembled; (**f**) bacterial cell lysis and release of new virus particles.

The lytic cycle consists of the following phases: (1) attachment: tail proteins bind to a specific bacterial cell surface receptor; (2) DNA injection: phage injects DNA genome through tail into bacterial cytoplasm; (3) DNA replication: bacteriophage DNA is reproduced in the bacterial cell, protein is synthesized and new capsids are formed; (4) bacteriophage assembly: capsids are filled with new DNA-forming phage particles; (5) lysis: genes poke holes along bacterial plasma membrane and cell walls so that water gets through and bacterium expands and bursts, releasing several new bacteriophages capable of infecting neighboring bacteria (Figure 2). 

Bacterial viruses are subdivided into 6 morphological groups according to phage components [11]: A—capsid and long tail with contractile sheath; B—capsid and long tail with rigid sheath; C—capsid and short tail; D—grand-size nucleocapsid with fibrous or spiky surface structures; E—phases incorporating one nucleocapsid; F—rod-like or filamentous phages.

Most bacteriophages of interest in oral cavity health belong to the order *Caudovirales.* Their capsid is composed of a protein coat and linear double-stranded DNA. Without an outer envelope, the head possesses cubic symmetry of a regular or elongated icosahedral type, and the tail forms a spiral. Capsomers are visible only under direct electron microscopy. The tail contains an end plate, pins and/or fibers for adsorption to the surface of bacterial cells. The genome of tailed bacteriophages usually consists of modules-interchangeable structural units. Genes responsible for similar functions are grouped into clusters [11].

The main advantages of phage therapy result from bacteriophage characteristics [10]: bacteriophages are highly strain-specific and have a low impact on the commensal flora; they multiply at the infection site and disappear with the target pathogen; a single application of bacteriophages may be highly effective; they are natural products devoid of apparent toxicity; they are easy to isolate and do not require complicated purification steps; they can be genetically engineered; they can destroy biofilm; and, as they are multiplying elements, their production is inexpensive. Its disadvantages, in contrast, are [10]: bacteriophages are strain-specific, which requires an accurate diagnosis before treatment; implementation of multispecies diseases treatment is more complicated; bacteriophages are immunogenic; temperate phages may carry and spread genes encoding toxins and/or antibiotic resistance determinants, limiting the number of possible phages for therapy; bacteriophages are dynamically evolving elements, which leads to regulation difficulties. 

## 3. Bacteriophages and Oral Health

A growing number of possible general applications of phage therapy in the oral cavity have been suggested. Bacteriophages are active against planktonic bacteria and, of greater interest for oral and dental treatments, against bacteria organized in biofilms. However, bacteria in biofilms may form anti-bacteriophage refuges, so that bacteriophages and bacteria may coexist [12]. Bacteriophages may change and adapt to target biofilm-embedded cells widespread in their ecosystem. Virions may access dense biofilm and spread through the tightly packed neighboring cells, weakening the whole structure. Additionally, some bacteriophages use various types of depolymerases to penetrate a bacterial capsule or biofilm matrix [13]. 

Some bacteriophages can be found in human saliva [14], and the most common hosts are Actinobacteria, Bacteroidetes, Firmicutes, Fusobacteria and Proteobacteria [15,16].

A recent study reviewed the main findings about bacteriophages and oral bacteria [17], which are summarized below.

- Actinomyces bacteriophages: They probably use surface structures that mediate the physical contact with streptococci as receptors. The interaction between streptococci and Actinomyces contributes to biofilm development [18]. Therefore, blocking co-aggregation with bacteriophages may reduce biofilm formation without eliminating health-associated Actinomyces, which may be used to control plaque development.
- Aggregatibacter bacteriophages: Aggregatibacter is implicated in localized aggressive periodontitis [19]. In vitro studies suggest that Aggregatibacter bacteriophages can transfer antibiotic resistance cassettes [20] and potentially increase release of leukotoxin [21]. The clinical impact of these findings remains uncertain.
- Enterococcus bacteriophages: Lysogeny has been observed in E. faecalis strains of oral origin [22]. Enterococci, occasionally involved in oral infections, may be controlled with a wide range of available bacteriophages, which may be especially helpful in cases of persistent endodontic lesions.
- Streptococcus bacteriophages: A diverse group of almost fifty bacteriophages of various morphotypes and both lifestyles may infect *S. mitis*, *S. mutans*, *S. oralis*, *S. salivarius* and *S. sobrinus* [23,24]. As streptococci are the primary colonizers in the formation of dental plaque, their use might prevent caries and periodontal disease.
- Fusobacterium bacteriophages: Fusobacterium nucleatum bacteriophages have been isolated from saliva samples [25]. However, lysis was slow.
- Porphyromonas, Prevotella and Tannerella: To date, only Prevotella bacteriophages have been detected in vivo [26]. More studies about these important anaerobic periodontopathogens should be conducted to define their possible clinical use.
- Treponema: To date, only one *T. denticola* bacteriophage has been detected [27].
- Veillonella: Only functional bacteriophages targeting Veillonella spp. have been described [28].
- Lactobacillus: Phages for the caries-associated Lactobacillus casei and six additional Lactobacillus species have been isolated [29].
- Lysins: Responsible for the safe, stable and easy production of phage enzymes that digest bacterial cell walls to liberate assembled phage particles. Recombinant lysins are often species-specific, and most are active against gram-positive bacteria. Lipopolysaccharides (LPS) protect gram-negative bacteria from lysins. Some lysins have been successfully tested against A. naeslundii and a broad range of Streptococcus species [30,31,32] and are, therefore, a novel type of antimicrobials that may be used to target oral bacteria.

Other phages have been described, but few have been isolated. The oral phage community is a potentially rich reservoir of therapeutic phages and enzymes, which have hardly been explored [17].

Some bacteriophages genetically engineered against Enterococcus faecalis biofilm have reduced the number of viable cells in vitro [33], and this finding adds to the current knowledge about the dental perspectives of phage therapy. 

## 4. Possible Uses of Bacteriophages in Dentistry

Dental caries: Isolation of Streptococcus mutans bacteriophages [23,24] is a possible use for the treatment of this disease. However, it has not been applied to clinical practice yet.

Periodontal diseases: Various aerobic and anaerobic pathogens are associated with periodontal diseases. The variations of bacteriophage communities in periodontal health and disease [34] and the fact that healthy individuals have a richer bacteriophage community suggest the possibility of developing therapies based on these communities. 

Endodontic lesions: Studies about the effect of bacteriophages on endodontic lesions have been restricted to Enterococcus faecalis [22,33]. The diverse microbial community of endodontic biofilms suggests that there may be room for further studies in this field. 

Periimplantitis: The identification of a bacteriophage that binds to the surface of zirconia well [35] suggests the existence of phages that may interfere with biofilms that cause periimplantitis; however, it is still early to test it clinically. 

Oral mucosal infections: A study has found that bacteriophage peptides may promote the proliferation of epithelial cells in the human oral mucosa without tumoral transformation [36], which might improve tissue healing. Phage therapy may advance rapidly in this field, because phage therapy for skin wounds has already been used for a long time. 

## 5. Discussion

Before antibiotics were discovered, there had been considerable research on bacteriophages as a treatment for human bacterial diseases. As bacteriophages attack only their host bacteria, not human cells, they are good candidates for this type of treatment.

After antibiotics were discovered, the study of bacteriophages was largely abandoned in many parts of the world. However, phages continued to be used for medical purposes in a number of countries, such as Russia, Georgia, and Poland, where they remain in use still today. As antibiotic-resistant bacteria have become much more prevalent, there has been increasing interest in bringing phage therapy back. 

The infective nature of several diseases—caries, periodontal diseases, periapical diseases, inflammatory disorders of the oral mucosa and infections due to implant procedures—suggests that specific bacteriophages may be used as aids to target bacteria in dentistry. Bacteriophages may destroy biofilm or limit its growth or maturation, which might reduce the impact of infections or control their acute phases. 

Phage therapy has some limitations, and one of them is the need to customize the treatment for each patient according to bacterial status. However, this may also be a virtue, as it targets only the bacteria that causes the disease. As the cost is low and the procedure to isolate a bacteriophage is straightforward, we envisage that more sensible procedures to prevent antibiotic resistance and enhance patient immunity will be developed in the future. 

## 6. Conclusions

After the rebirth of phage therapy, there has been room for various researchers to investigate this therapeutic alternative closely and to develop more conservative procedures as allies to current treatments. There should be a call for action in this field. Thus, dental surgeons should learn about bacteriophages.
